# Prevalence and Risk Factors of Anaplasmosis in Simmental Cattle in the Peruvian Amazon

**DOI:** 10.1155/2024/4634440

**Published:** 2024-06-19

**Authors:** Hugo Frias, Luis Murga, William Bardales, Vanessa Frias, Segundo Melecio Portocarrero-Villegas, Tatiana Segura Portocarrero, Miguel Arista, José Américo Saucedo-Uriarte

**Affiliations:** ^1^ Faculty of Zootechnical Engineering Agribusiness and Biotechnology of the Toribio Rodríguez de Mendoza National University of Amazonas, Chachapoyas 01001, Peru; ^2^ Laboratory of Infectious and Parasitic Diseases of Domestic Animals Livestock and Biotechnology Research Institute of the Toribio Rodríguez de Mendoza National University of Amazonas, Chachapoyas 01001, Peru

## Abstract

Anaplasmosis, transmitted biologically and mechanically, is one of the most prevalent diseases responsible for high production costs worldwide. In this research, the prevalence and risk factors of anaplasmosis in Simmental cattle raised in the Peruvian Amazon were evaluated. 266 blood samples were collected from bovines of different categories such as calves male, calves females, heifers <1.3 years, heifers >1.3 years, steers, bulls, and cows from the districts of Omia and Molinopampa. The enzyme-linked immunosorbent assay (ELISA) technique was used to detect antibodies against *Anaplasma marginale*. Of the 266 animals sampled, 67% were positive for A. marginale. A higher prevalence was determined in the district of Omia (99.3%), while in the district of Molinopampa, 28.7% was obtained. A prevalence of *A. marginale* was recorded in females (67.7%) and in males (64.8%) (*p* > 0.05). There is a significant association of the disease with the category of cattle, verifying the highest prevalence of *A. marginale* in calves male, heifer >1.3 years, and bull. The multiple correspondence analysis shows that San Mateo, Puma Marca, Mashuyacu, Primavera, and Los Olivos have a higher prevalence of anaplasmosis, associated with altitude of 1701–2000 m, spray baths and paddock rotation. Anaplasmosis is prevalent in Simmental cattle from the Peruvian Amazon, with a higher incidence in Omia and in females, considering May to August the critical months and the altitude less than 2000 meters above sea level.

## 1. Introduction

A series of vector-borne diseases have spread to new geographic areas around the world, and many of these diseases are caused by hemoparasites [1]. Diseases caused by parasites that develop in the blood (commonly called hemotropics) are transmitted by two routes, either mechanical or biological [2]. These parasites produce hematopic symptoms because they have an affinity for the bloodstream. The negative effects they cause in domestic animals are low weight gains, low levels of feed conversion, low meat and milk production, high costs in acquiring drugs, veterinary care, and mortality [1, 3]. Previous studies indicate that, in more than 70% of developing countries, it is affecting the economy of small and medium producers and therefore affecting food security [4].

Bovine anaplasmosis is caused by the bacteria *Anaplasma marginale*, and it is an infectious disease, ranging from acute to chronic [5]. This disease is transmitted by tick bites [6], or through blood transfer by external agents such as fly bites, blood-contaminated needles, ear markers, ear studs, and surgical equipment [7]. Ticks like *Rhipicephalus*, *Amblyomma*, *Hyalomma*, *Haemaphysalis*, *Dermacentor*, and *Ixodes* are responsible for transmitting common Anaplasma species such as *A. marginale* and *A. platys* in cattle [8]. Anaplasmosis has been reported in cattle, buffalo, sheep, goats, and some nondomestic ruminants [9–12]. The symptoms present in an animal sick with anaplasmosis are mainly progressive hemolytic anemia along with fever and jaundice, apathy, tachycardia, tachypnea, lethargy, decreased rumination movements, prostration, and hyperexcitability [7, 13].

Worldwide, 80% of cattle are affected by ticks and the transmission of their diseases [14]. Anaplasmosis in cattle has been causing morbidity and mortality, negatively impacting the producer's income due to reduced levels of milk and meat production [15]. For example, in Brangus cattle and their crosses, losses of US$34.61/animal have been reported in the initial phase of cattle and $7.97 per animal in the final phase of fattening [16]. Furthermore, among the risk factors that could be associated with anaplasmosis can be considered, the age of the livestock, the season of the year (summer), the breed, the size of the herd, the type of breeding, and the parasite infestation can be considered [17]. This research indicates that geographic region and sex are not important factors for the presence of *Anaplasma* [17]. However, there is a significant association between race and sex for the presence of anaplasmosis, while age is not an important factor [18]. In hot areas, *Anaplasma* sp. occurs due to the existence of influential factors such as the health status of the animal, environmental factors, agroecological conditions, and the cattle handling [19]. Furthermore, the prevalence rate is higher in autumn; likewise, they mention that the risk factors are type of breed (cross-breed), the load of ticks, and animals that cohabit with cattle (dogs) [20].

In Peru, one of the important sources of economic income is the raising of cattle. Furthermore, it is an important source of protein for its population. However, reports of diseases such as anaplasmosis in cattle are rare. That is why, in this research, the objective was to evaluate the risk factors and prevalence of anaplasmosis in Simmental cattle in the Peruvian Amazon.

## 2. Materials and Methods

### 2.1. Study Area

**Figure 1:**
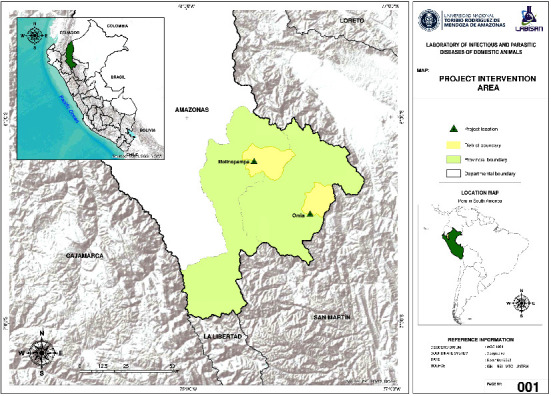
Geographical location of the experiment.

This research was conducted between June and November 2022 in the districts of Omia and Molinopampa, Amazonas region, Peru (Figure 1), located between 1300 and 2800 m.a.s.l. with average annual temperature between 15 and 30°C and average annual relative humidity between 72 and 92%.

### 2.2. Data Collection

The total population of Omia and Molinopampa was 5020 and 703 heads of cattle, respectively. It is important to note that this number of animals was before the beginning of the research [21]. The Simmental is a dual-purpose breed, steadily growing, and its grazing feeding systems are characterized by *Brachiaria decumbes*, *Pennisetum clandestinum*, *Dactilis glomerata*, and *Digitaria decumbes* with mineral supplementation.

For adjustment of the number of samples per stratum, we used the following formula:

(1) $$\begin{array}{c} n_{0}=\frac{n}{1+\left(n/N\right)\;}, \end{array}$$

where *n*_0_ is the sample size per stratum; *n* is the sample size; *N* is the number of heads of cattle per stratum.

The total sample was 144 for the Omia district and 122 for the Molinopampa district. The total coccygeal vein of 266 cattle (28 calves, 48 Calves females, 27 heifers <1.3 years, 24 heifers >1.3 years, 20 steers, 23 bulls, and 96 cows) was disinfected, and 3 ml of blood was obtained from each animal, in sterile Vacutainer® tubes without anticoagulant with sterile needles (Becton Dickinson Vacutainer). Centrifugation of the blood was performed at 1500 rpm for 10 minutes. The serum obtained was transferred to Eppendorf tubes, distributed in aliquots, and frozen at −20°C until analyzed by the indirect enzyme-linked immunosorbent assay (ELISA) test.

### 2.3. ELISA Test

The ELISA test was used to detect the presence of antibodies against *Anaplasma* spp. Polyvinyl 96-well plates were sensitized with recombinant MSP5 antigen (SVANOVIR® *A. marginale* – Ab, Svanova Biotech BA, Uppsala, Sweden), following the manufacturer's procedure. The reagents were equilibrated at room temperature before use.

In a proportion of 1 in 40 of PBS-twueen buffer, the predilution of each control was carried out. 100 *μ*l of each control (+ and −) and the prediluted samples in the wells of the microplates coated with the inactive noninfectious *Anaplasma* spp antigen were sealed and incubated at 37°C for 30 minutes. Subsequently, the microplates were washed four times with the PBS solution, and 100 *μ*l of the lyophilized conjugate was added, sealed, and incubated at 37°C for 30 minutes. The washing process described above was repeated and incubated at room temperature for 30 minutes. The reaction was interrupted by adding 100 *μ*l of stopping solution and mixing well. After 15 minutes of adding this solution, the optical density reading wavelength was established at 650 nm in a photometer (MRX Microplate Reader, DYNEX Technologies, Inc.). The interpretation and validity of the test were carried out following the recommendations of the kit manufacturer.

### 2.4. Questionnaire

Epidemiological data on possible risk factors were collected by applying a questionnaire to producers of each livestock herd at the time of taking blood samples. The information collected through the questionnaire covered variables that may be associated with the occurrence of a disease: altitude above sea level (1300 to 1700, 1701 to 2000, 2001 to 2300, and 2301 to 2800). In what months of the year have you observed that there are more cattle with anaplasmosis? (January-April, May-August, September-December, and does don't know). Do you have a health calendar? (Yes or No). Do you carry out any tick control measures? (Yes or No). If the answer is yes, what are the control measures? (internal and external antiparasitic, sprinkling baths, and paddock rotation or treatment when the disease occurs).

### 2.5. Data Analysis

The prevalence and association of anaplasmosis in Simmental cattle according to origin, sex, and animal category were analyzed with the chi-square test. Positive or negative values were applied to the variable's origin, sex, and category. The level of significance was set at *p* < 0.05. A multiple correspondence analysis (MCA) was applied to the values resulting from the questionnaire applied to the producers and to the positive or negative values resulting from the laboratory blood analysis. All analyses were performed in the statistical software R Studio vs. 4.3.3., based on the FactoMineR and factoextra libraries.

## 3. Results and Discussion

### 3.1. Prevalence

To diagnose anaplasmosis in cattle, 266 blood samples were analyzed and a 67% prevalence was found. A higher prevalence was recorded in the district of Omia (99.3%) than in Molinopampa (28.7%) (Table 1). An investigation in the Amazon of Ecuador determined that the province of Pastaza had a higher prevalence of anaplasmosis (65.51%) and the province of Napo registered 20%, being the lowest percentage [22]. Furthermore, in Brazil, a study mentions that the prevalence of anaplasmosis was 79.7% [23]. Prevalence results of 77.70% were reported [24], being higher to our findings in the district of Molinopampa. However, they were similar to the prevalence results of 27% recorded in the State of Santa Catarina [25]. Still, if we compare the results recorded in the Omia district, our findings exceed the values described by other studies. This district of Omia is characterized by being warm and humid, whose conditions are favorable for the development of ticks, which is stated by Kumar et al. [5], who reported a higher prevalence in tropical and humid places. The factors associated with the prevalence of anaplasmosis are the objective of breeding, tick control activities, medications used for tick control, and the category of livestock [26].

According to sex, prevalence was similar in female cattle (67.7%) than in male cattle (64.8%); therefore, no statistically significant differences were found (Table 1). In a study under similar feeding conditions, the prevalence of *A. marginale* in cows was 59.5%, which was lower than the values found in this study [27]. These results differ with the study by Sisson et al. [10], which mentions that males are more likely to be infected by *A. marginale* or *A. centrale*, due to the significant suppressive effect of testosterone on immune function in a variety of mammal species including cattle [28].

The anaplasmosis prevalence values found according to the category are shown in Table 1. There is a significant association between the disease and the category of cattle. There is a significant association of the disease with the category of cattle, verifying the highest prevalence of *A. marginale* in calves male, heifer >1.3 years, and bull. Previous studies report that *Anaplasma* affects young animals to a greater extent [10]. Animals younger than 1 year have a higher percentage of those affected by the disease (57.7%), compared to 41.7% in animals older than 4 years [26]. The high prevalence in calves is because tender animals have an undeveloped immune system and minimal exposure to diseases caused by parasites [5]. However, other research reports that adult animals tend to be positive 22 times more than young animals [29]. Such is the case of Vieira et al. [25], who indicate that the positivity of *A. marginale* is low in the age group of 24 to 36 months (7.69%) and 33.33% in younger animals (less than 6 months old). This could be due to the fact that in adult cattle, their immunity keeps the infection below detection levels [30]. Regarding the high prevalence in the bulls analyzed in this study, it could be due to the fact that males have a greater probability of being infected by *A. marginale* due to the significant suppressive effect of testosterone on immune function in various mammal species [10, 28].

**Figure 2:**
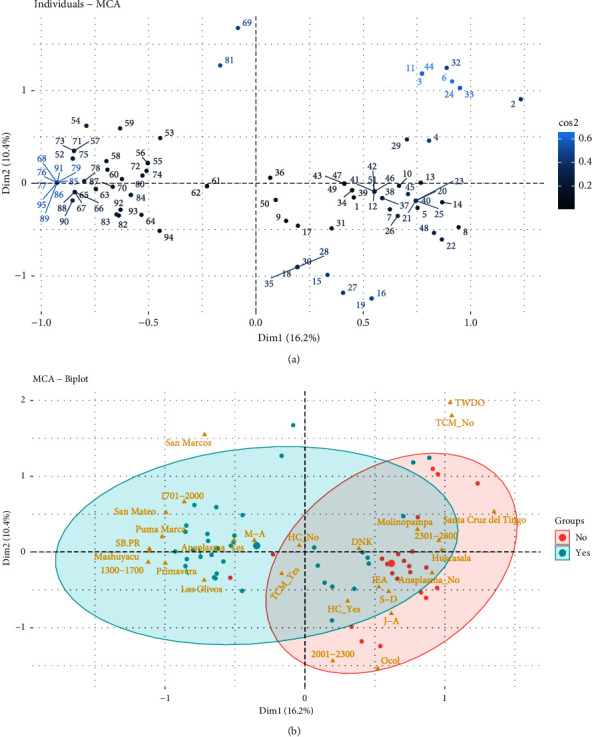
Multiple correspondence analysis. (a) Distribution of individuals expressed in Cos2. (b) Biplot of the level of association of anaplasmosis according to the sampled site.

Figure 2(a) shows the distribution of the surveyed individuals, obtained from multiple correspondence analysis (MCA). Here, it is shown that each point represents a surveyed individual and are projected in two dimensions (Dim 1 and Dim 2), which explain 16.2% and 10.4% of the total variability, respectively. Furthermore, the total variance explained between two components was 26.6%. The points present in the figure indicate the individual observations and are colorcoded (blue color, presence of anaplasmosis and red color, absence of anaplasmosis) (Figure 2(b)). In this research, San Marcos, San Mateo, Puma Marcos, and Mashuyacu are strongly associated with the presence of anaplasmosis. There are areas where the presence of the disease is permanent, and they are those where the climatic conditions are favorable for the growth and survival of the vectors, so the parasite persists throughout the year, leaving offspring [31]. In these areas, animals develop resistance to anaplasmosis; however, they can become infected by having a weakened immune system, so when they are moved to areas with a prevalence of ticks, where anaplasmosis is present and the type of husbandry is intensive, there is the possibility that the health of adult animals will be affected and calves [32]. On the other hand, Santa Cruz del Tingo and Ocol show an association with the absence of the disease. However, Molinopampa and Huascasala may or may not be associated with anaplasmosis because they are shown sharing the ellipses.

**Figure 3:**
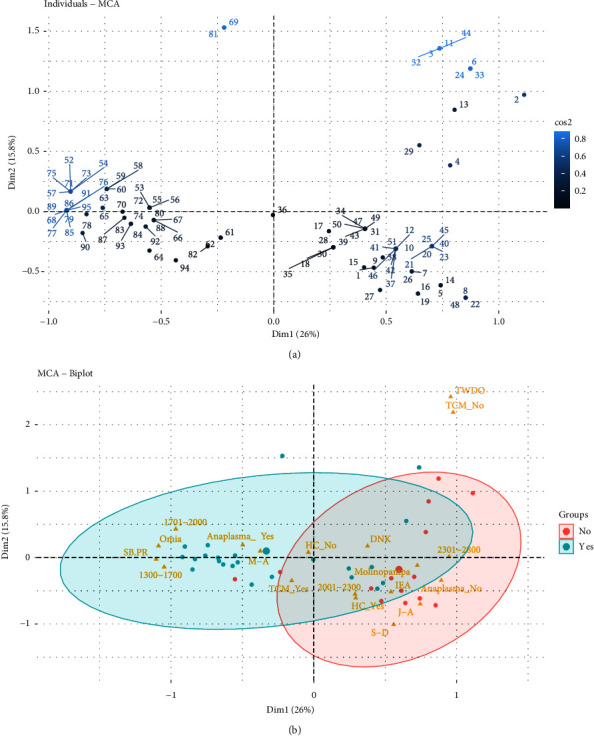
Multiple correspondence analysis. (a) Distribution of individuals expressed in Cos2. (b) Biplot of the level of association of anaplasmosis according to the sampled district.

When performing the MCA considering the district (Omia and Molinopampa), the two main dimensions (Dim1 and Dim2) jointly explain 41.8% of the total variability in the data (Figure 3). The Omia district presents greater susceptibility to the risk of anaplasmosis compared to the Molinopampa district. It is observed that the lower the altitude above sea level is associated with anaplasmosis and altitudes from 1300 to 2000 meters are associated with this disease (Figure 3(b)). Likewise, sprinkling baths and paddock rotation (SB-PR) and May-August (M-A). Control practices such as spray baths are aimed at controlling the tick vector; however, in this study, the APC analysis shows that it has no effect on the control of Anaplasma marginal. Furthermore, the practice of rotating pastures is frequent; however, the pastures of the Omia district have a high prevalence of ticks because the incubation period of this parasite is not shortened. In previous research, it has been reported that the season of the year can be considered a risk factor, with the summer months being the increase in prevalence [17, 18]. Coinciding with our findings, the geographical region of tropical humid forest is the area of greatest risk for the prevalence of anaplasmosis. In addition, the Omia district is within the tropical humid zone, with greater precipitation in the months of March to April. Although it has also been reported that in hot areas, *Anaplasma* sp., it occurs due to the existence of influential factors such as the health status of the animal, environmental factors, agroecological conditions, and cattle handling [19]. Furthermore, the prevalence rate is higher in autumn; likewise, they mention that the risk factors are the load of ticks and animals that cohabit with cattle (dogs) [20].

The months that do not show a risk of anaplasmosis are January to April (J-A) and September to December (S-D). In addition, the variables that are and are not associated with anaplasmosis are use and not of the health calendar (HC_No and HC_Yes), altitude from 2001 to 2300 meters above sea level, and internal and external antiparasitic (IEA) (Figure 3(b)). This difference may be due to the adequate control of arthropods that act as vectors of the disease, promoting contagion [33]. However, the sampling time can influence the results since studies indicate that the highest values of *A. marginale* infection are recorded in the summer [34]. Furthermore, the lower the rainfall, the lower the availability of forage, and the tree cover and the number of vectors such as ticks and flies increase, creating a favorable environment for their reproduction [35]. In lowlands at 2000 meters above sea level, susceptibility has been found because favorable conditions are created for the development and reproduction of vectors such as ticks [31].

Although in our research, the variable treatment when the disease occurs (TWDO) and not having tick control measures (TCM-No) are shown outside the two groups formed (presence or absence of Anaplasmosis), it has been reported that, when it is determined that an animal is positive for anaplasmosis, the treatment is carried out based on antibiotics that contain oxytetracycline and chlortetracycline in different compositions and with different adjuvants in their formula, which promote an antibiotic reaction against the bacteria, ensuring a rapid recovery of the animal [22].

## 4. Conclusions

Anaplasmosis is a disease that affects cattle worldwide, and it develops in areas where climatic conditions such as temperature and humidity encourage the spread of the vector. The district of Omia presented 99.3% prevalence, while the district of Molinopampa presented 28.7%. Likewise, no differences were found in the prevalence of *A. marginale* according to sex, being 64.8% in males and 67.7% in females. Of the categories evaluated, a significant association of the disease with the category of cattle was recorded, being higher in male calves, heifers >1.3 years old, and bulls.

The multiple correspondence analysis indicates that San Mateo, Puma Marca, Mashuyacu, Primavera, and Los Olivos are associated with a higher prevalence of Anaplasmosis. The altitude of 1701 to 2000 meters above sea level, sprinkling baths, and paddock rotation and May–August showed a risk factor for the presence of anaplasmosis disease.

## Figures and Tables

**Table 1:** Relative frequency (%), positive and negative cases (*n*) for anaplasmosis diseases according to origin and sex.

	Positive		Negative		Mean	

Anaplasmosis						
Omia	99.3%	(143)	0.7%	(1)	54.1%	(144)
Molinopampa	28.7%	(35)	71.3%	(87)	45.9%	(122)
*p* value^1^	<0.05					
Sex						
Male	64.8%	(46)	35.2%	(25)	26.7%	(71)
Female	67.7%	(132)	32.3%	(63)	73.3%	(195)
*p* value^1^	0.656					
Category						
Calves male	78.6%	(22)	21.4%	(6)	10.5%	(28)
Calves female	56.3%	(27)	43.8%	(21)	18.0%	(48)
Heifer	63.0%	(17)	37.0%	(10)	10.2%	(27)
Heifer >1.3 years	79.2%	(19)	20.8%	(5)	9.0%	(24)
Steers	30.0%	(6)	70.0%	(14)	7.5%	(20)
Bull	78.3%	(18)	21.7%	(5)	8.6%	(23)
Cow	71.9%	(69)	28.1%	(27)	36.1%	(96)
*p* value^1^	0.002					

^1^The *p* value indicates the asymptotic (two-sided) significance of the association.

## Data Availability

All data pertaining to the current study are available from the corresponding author upon a reasonable request.
